# Plant Cation-Chloride Cotransporters (CCC): Evolutionary Origins and Functional Insights

**DOI:** 10.3390/ijms19020492

**Published:** 2018-02-06

**Authors:** Sam W. Henderson, Stefanie Wege, Matthew Gilliham

**Affiliations:** ARC Centre of Excellence in Plant Energy Biology, School of Agriculture, Food and Wine, University of Adelaide, PMB1, Glen Osmond, SA 5064, Australia; sam.henderson@adelaide.edu.au (S.W.H.); stefanie.wege@adelaide.edu.au (S.W.)

**Keywords:** KCC, NKCC, plant membrane transport, plant nutrition, anion, Golgi, plasma membrane, AT1G30450, Arabidopsis

## Abstract

Genomes of unicellular and multicellular green algae, mosses, grasses and dicots harbor genes encoding cation-chloride cotransporters (CCC). CCC proteins from the plant kingdom have been comparatively less well investigated than their animal counterparts, but proteins from both plants and animals have been shown to mediate ion fluxes, and are involved in regulation of osmotic processes. In this review, we show that CCC proteins from plants form two distinct phylogenetic clades (CCC1 and CCC2). Some lycophytes and bryophytes possess members from each clade, most land plants only have members of the CCC1 clade, and green algae possess only the CCC2 clade. It is currently unknown whether CCC1 and CCC2 proteins have similar or distinct functions, however they are both more closely related to animal KCC proteins compared to NKCCs. Existing heterologous expression systems that have been used to functionally characterize plant CCC proteins, namely yeast and *Xenopus laevis* oocytes, have limitations that are discussed. Studies from plants exposed to chemical inhibitors of animal CCC protein function are reviewed for their potential to discern CCC function in planta. Thus far, mutations in plant *CCC* genes have been evaluated only in two species of angiosperms, and such mutations cause a diverse array of phenotypes—seemingly more than could simply be explained by localized disruption of ion transport alone. We evaluate the putative roles of plant CCC proteins and suggest areas for future investigation.

## 1. Introduction

Cation chloride (Cl^−^) cotransporters (CCC) are membrane-integral solute carriers that mediate electroneutral translocation of Cl^−^, coupled to potassium (K^+^) and/or sodium (Na^+^). Belonging to the Solute Carrier 12 (SLC12) family of proteins, CCC members are present in plants, animals, fungi and prokaryotes [1]. It has been previously shown that the SLC12 family consists of up to four subfamilies including NKCC/NCC, KCC, CCC9 and CCC interacting protein (CIP9) [1]. Phylogenetic analyses have revealed that all plant CCC proteins belong to the KCC subfamily of CCCs [1,2,3]. In mammals, mutations in CCC proteins can cause diseases such as Gitelman syndrome, affecting kidney function [4], and neurological disorders [5]. To date, plant CCC transporters have been cloned and functionally characterized from Arabidopsis (*Arabidopsis thaliana*) [3], rice (*Oryza sativa*) [6] and grapevine (*Vitis vinifera*) [7]. Disruption of genes that encode CCC proteins in plants leads to severe growth and developmental phenotypes [3,6]. However, compared to CCC proteins from animals, CCC proteins from plants have been studied in much less detail. Therefore, many questions regarding plant CCC function remain unanswered. For example, can the phenotypes of plant *ccc* mutants be explained simply by disrupted ion transport? Are there other in planta functions of plant CCC proteins that are yet to be revealed? This review describes what is currently known about CCC proteins in plants, investigates their evolutionary origins and phylogenetic relationships, evaluates the current methods used for characterizing plant CCC proteins, and describes avenues for future investigations to broaden our understanding of this unique class of plant transporter.

## 2. Evolution of Plant CCCs

Cation-chloride cotransporters have been identified in all kingdoms including prokaryotes and eukaryotes [1]. However, the diversity of CCCs within the plant kingdom has not been thoroughly examined. Table 1 summarizes *CCC* genes identified in a selection of plants with sequenced genomes. Putative orthologues of Arabidopsis AtCCC1 (At1G30450) were obtained from Ensembl Plants [8], and polyploid species were excluded. The genome of the basal angiosperm *Amborella trichopoda* encodes a single *CCC* gene, and the number of *CCC* genes per genome of eudicots is generally one or two. Selected diploid monocots with sequenced genomes have two or three *CCC* genes per genome. To date, chlorophyte (green algae) genomes appear to contain either none or a single gene. It is unknown whether these numbers will remain consistent as well-annotated genome sequences of more species are revealed, but an absence of *CCC* genes from selected chlorophytes would demonstrate that *CCC* genes are not crucial for the survival of some single-celled species. Worden et al. [9] compared the genomes of two species of the genus *Micromonas*, marine algae, with two other marine algal species of the genus *Ostreococcus*. In that study, the genomes of the *Micromonas* spp. were shown to contain a single *CCC* gene each, whereas the *Ostreococcus* spp. lacked a *CCC* in their genomes [9]. Like *Ostreococcus* spp., the model organism and freshwater algae *Chlamydomonas reinhardtii* lacks a *CCC* in its genome (Table 1). The observation that land plants possess *CCC* genes, and only some green algae contain *CCC*s while others do not, suggests that *CCC* genes were present in the last common ancestor of green algae (chlorophytes) and land plants (streptophytes) [10]. Unlike chlorophytes, all investigated streptophytes contain at least one CCC in their genome (Table 1). Surprisingly, the genome of the bryophyte and model organism *Physcomitrella patens* encodes seven *CCC* genes (Table 1).

**Figure 1:**
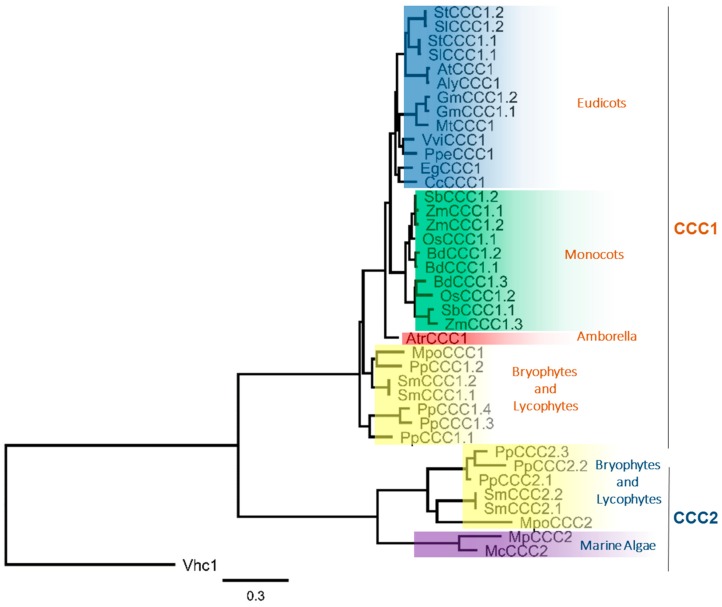
Phylogenetic relationship of the plant CCC family shows two distinct clades. Maximum likelihood tree rooted to yeast CCC “Vhc1”. Generated using MUSCLE alignment, Gblocks curation followed by PhyML phylogeny (www.phylogeny.fr) [15]. See Table S1 for accession numbers.

Using the predicted protein sequences encoded by the *CCC* genes identified in Table 1, we prepared a phylogenetic tree (Figure 1). The phylogenetic analysis revealed that plant CCC proteins form two distinct clades, that we have denoted CCC1 and CCC2. These two distinct phylogenetic branches have previously been identified in the moss *P. patens*, and both clades were shown to be phylogenetically related to the KCC subfamily [2]. We identified the CCC2 clade to be present only in lycophytes, bryophytes and algae (Figure 1). *P. patens* contains members from both families, leading to the large number of *CCC* genes in the *P. patens* genome. The CCC2 family is not present in vascular plants beyond the lycophyte lineage, while the investigated chlorophytes do not harbor members of the CCC1 clade, and angiosperms contain exclusively CCC1 family members (Figure 1). Thus evolutionarily, it appears that gene duplication events have occurred at the base of the streptophytes, leading to the formation of two distinct clades, with some species having many paralogs (e.g., *P. patens*). Later in evolution, it is likely that gene-loss events have occurred, leading to an absence of *CCC2* genes in angiosperms. Fewer copies of *CCC1* genes in eudicots compared to monocots, could result from gene loss in eudicots, or a second duplication event in monocots. Such events could arise in plants during DNA replication and recombination, unequal crossing over during meiosis, transposable elements that allow for transduplication or retropositioning, or the accumulation of mutations leading to non-functionalization [11,12]. Whole genome duplication and polyploidization in plants would lead to even greater copies of *CCC* genes. Addressing such questions will become practicable with the continued sequencing and annotation of complex genomes from additional plant species such as *Triticum aestivum* (wheat) [13] and *Chenopodium quinoa* (quinoa) [14]. The observation that SmCCC1.1 and SmCCC1.2 from *Selaginella moellendorffii* only differ by a single amino acid residue strongly suggests that gene duplication is the reason for multiple CCC1 copies in that species. Gene duplication could also be the cause of multiple *CCC* genes in some other vascular plant species, which raises the possibility of redundancy and a release from selective pressure on one or more of the *CCC* paralogs. It is noteworthy that rice, which contains two *CCC* paralogs in the genome (*OsCCC1.1* and *OsCCC1.2*), displayed a reduced growth phenotype when only *OsCCC1.1* was mutated, and *OsCCC1.2* remained intact [6]. It is possible that some *CCC* genes, in plants with multiple *CCC* paralogs, are non-functional but this remains to be tested.

**Figure 2:**
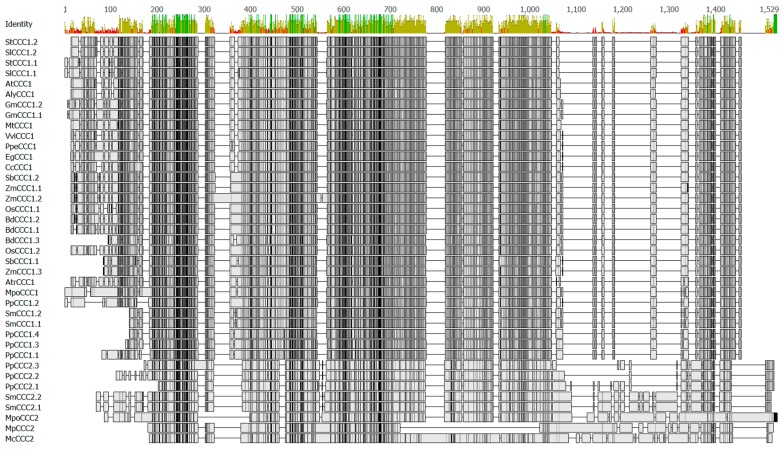
Amino acid sequence alignment of selected plant CCC proteins from Table 1 and Figure 1. Alignment was generated using Clustal Omega [16] and edited using Geneious R8 (Biomatters). Percent identity is shown above the alignment (green 100%, brown 30% to 99%, red < 30%). Similarity shading is: black 100% similar; dark grey 80% to 99% similar; light grey 60% to 79% similar and white <60% similar. For list of protein accession numbers, see Supplementary Table S1. For a percent identity matrix, see Table S2.

Comparing the functional properties of proteins from the CCC1 and CCC2 clades would be interesting to determine whether subfunctionalization or neofunctionalization occurred within the plant *CCC* gene family. There are regions of interest within primary structures of CCC proteins that could be used to probe for functional differences between CCC1 and CCC2 clades, and these are shown in Figure 2. Compared to proteins of the CCC2 family, the CCC1 proteins have a shorter amino (N)-terminus, and an insertion (with low percent identity) towards the carboxyl (C)-terminus (Figure 2, residues 1050 to 1350). Plant CCC proteins are long polypeptides of approximately 700 to 1200 amino acids in length (Figure 2), and have been predicted to possess large hydrophilic C-termini [3,7]. How these features affect the structure, function, and regulation of CCC proteins is unknown.

## 3. Putative Roles for CCC in Plant Growth and Development

**Figure 3:**
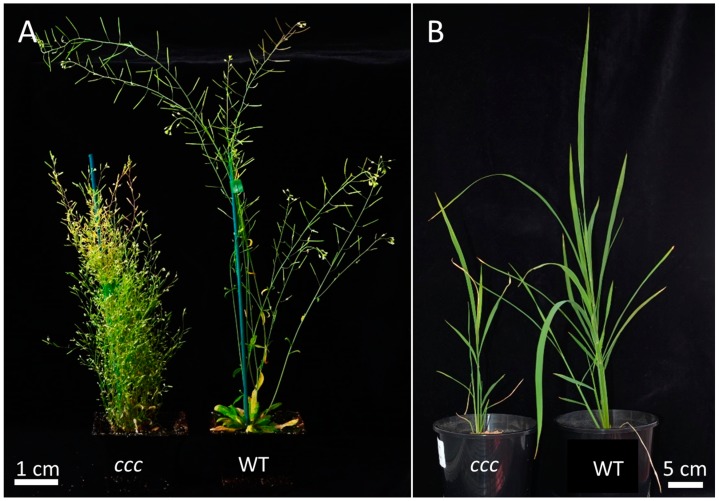
Phenotypes of mature Arabidopsis (**A**); and rice (44 days after sowing) (**B**) *ccc1* knockout plants compared to their respective wildtypes. The Arabidopsis knockout displays a complete loss of apical dominance, dwarfed growth, shorter siliques and reduced seed set [3], while the rice *ccc* displays dwarfed growth and a reduced leaf blade size [6].

Phenotypes of *ccc* loss-of-function mutants have been described in two plant species—Arabidopsis and rice. In both species, knockout mutants show an array of altered anatomical and physiological aspects and severely reduced fitness [3,6]. Directly connecting the function of the protein (i.e., ion transport) to the various phenotypic alterations in the knockout mutants is not straight forward. In general, plant CCC1 proteins seem to have a common function in different species, as constitutive ectopic expression of the grapevine *VviCCC1* in the Arabidopsis *ccc1* mutant was able to complement the knockout phenotype [7]. Whether a monocot CCC1 family member, or a CCC2 family member, is also able to rescue the Arabidopsis *ccc1* mutant would be interesting to test. In both Arabidopsis and rice, loss of a known functional CCC1 leads to stunted shoot growth (Figure 3) and short roots [3,6]. Furthermore, rice *ccc* roots are smaller in diameter and consist of smaller cells [6], while this has not yet been characterized in Arabidopsis. Arabidopsis *ccc* plants display a complete loss of shoot apical dominance, are heavily branched, and the emerging inflorescence stems frequently develop necrosis in the elongation zone [3]. This might indicate a role for CCC1 in rapidly elongating cells, for example for turgor establishment or regulating delivery of lipids and/or cell wall material to the plasma membrane. The loss of apical dominance might be connected to this frequent necrosis in the elongation zone, or possibly to a failure to establish an auxin gradient through a yet unknown mechanism. Disrupted auxin gradients are responsible for dwarf and bushy phenotypes in other Arabidopsis mutants such as *echidna* (*ech*) [17,18], *myosin 2* (*mya2*) [19] and *weak auxin response1* (*wxr1*) [20]. How mutation of an inorganic ion transporter in *ccc1* mutants confers such a phenotype is therefore unclear.

The first analysis of *CCC1* gene expression patterns was performed in Arabidopsis using a 723 base pair (bp) promoter: β-Glucuronidase (GUS) construct. These experiments indicated that AtCCC1_pro_ drives GUS expression in specific tissues and cells types, with a high level of expression in the root tip, vasculature, stipules, hydathodes, stamen and pollen, while no expression was detected in the shoot meristem, in guard cells or epidermal cells [3]. This contrasts with the wide impact of the loss-of-function on the plant, and with the variety of phenotypic aspects observed in many different plant organs. Arabidopsis *CCC1* was not present on the first generations of most microarray chips; however, some more recent studies using RNAseq in Arabidopsis show that *AtCCC1* is expressed in root hairs and root epidermis [21], which demonstrates that *AtCCC1* is expressed in other tissue types besides those detected by the GUS reporter system.

In grapevine, tissue specific expression analysis by semi-quantitative RT-PCR revealed that *VviCCC1* is expressed in all tissue types tested, including flowers, berries, leaves and roots [7]. In rice, tissue specific expression analysis by quantitative real-time PCR (qPCR) revealed *OsCCC1.1* expression in leaves and very high expression in the root tip [22]. Furthermore, OsCCC1.1-promoter (2.5 kb) driven expression of the OsCCC1.1 protein C-terminally tagged with GFP revealed additional expression in all root cell types [6]. In that study, antibodies raised against a short peptide (corresponding to residues 870 to 887 of OsCCC1.1), indicated protein presence in the root tip and in all leaf cells [6]. This is in agreement with qPCR studies of *PtrCCC* from trifoliate orange (*Poncirus trifoliata*) root tips [23]. Expression of *AtCCC* in stamens was found in an early study searching for genes essential for correct gametophytic function [24]. In that study, AtCCC1 was named HAP5 (for hapless), and the *ccc1* allele showed reduced transmission in both female and male gametophytes in the *quartet1* pollen mutant background [24]. AtCCC1 function is therefore important for pollen development, and for correct function or development of the embryo sac [24]. How exactly the ion transporter influences all these aspects remains unknown.

Some of the CCC proteins in mammals are associated with osmoregulation in organs such as kidneys, for example the human hNKCC2 [4]. A similar function (i.e., cellular osmoregulation) might also be possible in plants. Rice OsCCC1.1 is important for reaching optimal root osmolality (cell sap, xylem and phloem content not separated) [6], while Arabidopsis AtCCC1 and grapevine VviCCC1 might play a role in ion homeostasis, which might include adjustment to increased osmolality when salts are applied to the growth medium [3,7]. However, the complex phenotype of Arabidopsis knockout plants suggests that osmotic adjustment, if AtCCC1 plays a role here, is not the only cause. Interestingly, both AtCCC1 and VviCCC1 are localized to the Golgi and *trans*-Golgi network (TGN), and fluorescent fusion proteins are not readily detectible on the plasma membrane [7,25]. It is possible that CCC only localizes to the plasma membrane in specific cell types or under specific conditions, like for example the Arabidopsis iron transporter IRT1 [26], or that CCC is involved in processes in these organelles, such as vesicle formation or cargo selection. Conversely, rice OsCCC1.1 is possibly additionally localized on the plasma membrane [6].

## 4. Influence of CCC Proteins on Plant Ion Homeostasis

Chloride is a component of salt stress when plants are exposed to high salt concentrations [27], and an important plant macronutrient at lower salt concentrations [28]. As Cl^−^ is one of the known substrates of CCC proteins from both plants and animals, some studies have investigated CCC proteins to determine their role in Cl^−^ homeostasis [2,7,22]. One study ectopically expressed *PtrCCC* from trifoliate orange in stably transformed *Nicotiana nudicaulis* using the constitutive CaMV35S promoter [23]. In that study, less Cl^−^ was present in roots and shoots of transgenic lines under KCl stress compared to wild-type [23]. However, in a comparison of the closely related Cl^−^-including and Cl^−^-excluding *Citrus* spp. rootstocks (Cleopatra mandarin and Carrizo citrange, respectively), *CcCCC* was not found to be transcriptionally regulated by Cl^−^ treatment. This was also the case when *CCC* expression was compared between *Vitis* spp. rootstocks with contrasting Cl^−^-exclusion capacities under control and mixed Cl^−^ treatments by microarray hybridization [29] and qPCR [7]. The level of expression of *PtrCCC* in transgenic *N. nudicaulis* relative to the endogenous *N. nudicaulis CCC* was not determined [23], but presumably the transgenic tobacco lines would have expressed *PtrCCC* at a greater abundance than natively in *P. trifoliata*. The mechanism for reduced root and shoot Cl^−^ in transgenic tobacco is unknown. In another study using *Lotus* as a reference, and assuming a stoichiometry of 1K^+^:1Na^+^:2Cl^−^, it was concluded that transport would most likely be towards the xylem if the CCC transporter resided on the plasma membrane of root xylem parenchyma cells [30]. Hence, it is unlikely that CCC proteins contribute to the role of xylem parenchyma cells as gatekeepers for xylem retrieval of potentially toxic Na^+^ and Cl^−^ ions [31]. Conclusions that plant CCC proteins are responsible for salt tolerance must therefore be carefully considered. In Citrus, grapevine and probably in some other plant species, *CCC* genes are not transcriptionally regulated, but they might be regulated post-transcriptionally by phosphorylation. AtCCC has been identified as a putative mitogen activated protein kinase 6 (MPK6) phosphorylation target [32]. Phosphorylation and de-phosphorylation of animal NKCCs drastically changes the NKCC ion transport properties [33]. Further studies are required to investigate the regulation of plant CCCs.

## 5. Water as a Putative Substrate of Plant CCC Proteins

The concept that proteins in the plasma membrane of plant cells might simultaneously transport ions and water has existed for some time [34]. In the water-cotransport scenario, the net free energy change of water and ion cotransport could be thermodynamically favorable despite unfavorable thermodynamics for water movement by itself. Several studies in mammals have identified CCC proteins that have water and ion cotransporter activity. Using simultaneous swelling and tracer flux experiments in oocytes from the African clawed toad *Xenopus laevis*, mouse (*Mus musculus*) NKCC2 was shown to transport 460 water molecules for each turnover of the protein [35]. These observations have led to the suggestion that CCC proteins from vascular plants could have a similar function, and be responsible for ion-coupled water transport—providing a molecular mechanism for phenomena such as root pressure gradients and embolism repair in xylem vessels [36,37,38,39]. This is a possible scenario, especially since an Arabidopsis aquaporin (AtPIP2;1) was recently shown to permeate both water and sodium ions [40]. The energy consumption for night-time filling of root xylem vessels of barley via a water-ion cotransport mechanism under 12 h light/dark cycle, was calculated to be 0.0135% of the energy provided through photosynthesis [41]. In that study, it could not be excluded that water cotransport in plants contributes significantly to xylem filling during night-time transpiration [41]. However, at present, there is insufficient evidence to conclude that CCC proteins have a role in ion-coupled water movement.

Ion-coupled water transport into the root xylem is unlikely to be the exclusive role of CCC proteins in planta, because (as mentioned in Section 3) AtCCC1 promoter GUS expression and RNA-sequencing revealed expression in many tissues types, and not exclusive expression in the vasculature [3]. Furthermore, transcripts encoding *OsCCC1.1*, *VviCCC1* and *PtrCCC1* show variable expression patterns in many different root and shoot tissues [6,7,22,23]. Further experiments are indeed required to determine whether plant CCC1 and CCC2 proteins, like mouse NKCC2, are dual water ion cotransporters [39].

## 6. Using Pharmacology to Gain Insight into Plant CCC Transport Function

Loop diuretics are pharmaceutical compounds that are often used to treat hypertension in humans. The loop diuretics furosemide, thiazide and bumetanide achieve this function by acting as blockers of CCC proteins. These synthetic compounds function as competitive inhibitors at the Cl^−^ binding site of the cotransporter. Furosemide preferentially inhibits KCCs, thiazides inhibit NCCs, while bumetanide inhibits NKCCs. In addition to its effect on mammalian CCC proteins of the NKCC class, bumetanide seems to have a direct pharmacological blockage effect on plant CCC proteins expressed in *X. laevis* oocytes [3]. However, the information gained about plant proteins in an animal expression system is limited, as animal cells are inherently sensitive to loop diuretics themselves (*X. laevis* oocytes express their own CCCs, described in Section 7.1). Thus, what can we infer from the application of these inhibitors to plant cells in vivo?

Germination of tobacco pollen grains was weakly inhibited (<50%) by furosemide and bumetanide treatment [42]. Since the effect of the inhibitors on germination was only achieved at very high concentration (1 mM), it was concluded to most likely be nonspecific [42]. These results indicate that CCC proteins are inaccessible to the applied inhibitor or they might not be required for pollen germination. This is consistent with the phenotype of Arabidopsis *ccc* (*hap5*) pollen, which developed poorly but developed pollen were still able to germinate and fertilize the female gametophyte [24]. Hence, the in planta function of CCC in reproduction may predominately occur before pollen germination.

Bumetanide has been demonstrated to act as a plant defense elicitor. When applied at 100 µM to suspension cells or roots of whole seedlings, bumetanide stimulated pathogen induced cell death, which enhanced the resistance to *Pseudomonas syringae* pv. Tomato [43]. It is tempting to speculate that this immune-priming of Arabidopsis to *P. syringae* arises through inactivation of CCC proteins by bumetanide. However, numerous other synthetic compounds, with no known CCC inhibitory activity, had similar immune priming effects on Arabidopsis [44,45,46]. Therefore, more research is required to uncover the mode of action of bumetanide in plant immunity.

CCC inhibitors, including bumetanide, also affect ion fluxes in root cells of whole plants. Arabidopsis root cells have been shown to have a fast acting, passive Cl^−^ influx upon exposure to salt, followed by a slower Cl^−^ influx phase most probably mediated by active or secondary active transport. In one study, salt-induced Cl^−^ influx into cells of the root-hair-zone in Arabidopsis seedlings during the active uptake phase was inhibited by 100 µM bumetanide to around 40% [47]. Compatible with this, Na^+^ and K^+^ efflux from isolated barley root stelar tissue (from three-day-old seedlings) was inhibited in the presence of 100 µM bumetanide [48]. While this inhibition of influx and efflux could be due to blocking the Na^+^-K^+^-Cl^−^ cotransport of the CCC at the plasma membrane, the localisation of AtCCC1-GFP to endomembranes in tobacco leaves and Arabidopsis protoplasts [7], as well as proteomic data [49,50] would suggest a different mechanism. One alternative is that bumetanide blocks the CCC-mediated trafficking of other anion and cation channels to the plasma membrane, or the delivery of vesicles containing ions to the plasma membrane for extracellular release. This might explain why other anion channel blockers like 4,4′-diisothiocyanatostilbene-2,2′-disulfonic acid (DIDS) had a much greater inhibitory effect on salt-induced root Cl^−^ influx of Arabidopsis compared to bumetanide [47].

## 7. Useful Expression Systems to Functionally Characterize Plant CCC Proteins

Various heterologous expression systems have been used to functionally characterize mammalian CCC proteins, either alone or co-expressed with an interacting partner (e.g., protein kinases). Some of these expression systems have been applied to the study of plant CCC proteins.

### 7.1. Xenopus laevis Oocytes

Plant CCC proteins reach the plasma membrane when expressed in *X. laevis* oocytes [7]. This enables net ion fluxes through CCC to be directly measured. In oocytes expressing AtCCC1, the net uptake of radiotracers ^22^Na, ^86^Rb (for K^+^) and ^36^Cl was measured by scintillation counting, and results indicated that AtCCC1 mediates uptake of all three ions [3]. Similar results were found when this technique was applied to investigate the substrates of VviCCC1 [7]. Although these results are indicative of plant CCC proteins having an inorganic ion cotransporter function, a few complications of the *X. laevis* oocyte expression system for plant CCC proteins should be noted. Firstly, the activity of plant CCC proteins expressed in *X. laevis* oocytes is much lower compared to the activity of exogenously expressed mammalian CCCs in this system. Secondly, oocytes themselves harbor endogenous electroneutral KCC and NKCC proteins with quite high activities [51,52]. Additionally, the ^86^Rb-uptake activity of the endogenous bumetanide-sensitive NKCC of *X. laevis* oocytes is highly dependent on season, and animal housing and husbandry [53]. Thus, the radiotracer uptake activity of *X. laevis* oocytes expressing a plant CCC (relative to non-expressing controls) could show variations across batches, seasons or even laboratories. Silencing the endogenous *X. laevis CCC* cannot be considered a viable option because the cellular machinery required for RNA-induced gene silencing is not fully developed in *X. laevis* oocytes [54]. Thus, alternative mammalian expression systems may help to elucidate the functional properties of plant CCC proteins. A low K-resistant epithelial mutant cell line, derived from dog kidney, has previously been demonstrated to have little bumetanide-sensitive ^86^Rb, ^22^Na, or ^36^Cl uptake (<2% of control) [55]. This cell line, known as Madine Derby canine kidney (MDCK) LK-C1, was successfully employed to study human hNKCC1 [56], and may provide an alternative to *X. laevis* oocytes and potentially be a promising tool for the study of plant CCC proteins.

### 7.2. Yeast

Yeast (*Saccharomyces cerevisiae*) is a useful expression system for characterizing plant ion transporters with cation selectivity [57], and has been used for such proteins as the High Affinity K^+^ Transporters (HKT) [58] and the inward rectifying K^+^ channel KAT1 among other proteins. OsCCC1.1 complemented the K^+^ uptake deficient (*trk1Δ trk2Δ*) mutant CY162 [6], suggesting that OsCCC1.1 is capable of K^+^ uptake in yeast. Similarly, when expressed in the mutant yeast strain G19, which is deficient in Na^+^ and K^+^ plasma-membrane efflux systems (*ena1-5Δ nha1Δ*) and sensitive to high Na^+^ concentrations, OsCCC1.1 exacerbated the phenotype [6], which suggests Na^+^ uptake through OsCCC1.1. Interestingly, overexpression of hNKCC2 had the opposite effect of OsCCC1.1 on a Na^+^ sensitive yeast strain—hNKCC2 improved the growth of BYT45 (*ena1-5Δ nha1Δ*) on media containing high KCl and NaCl concentrations [59]. This opposite effect cannot be explained yet, but might be due to different transport directions or different subcellular localizations of human and plant CCC proteins in yeast cells. The yeast CCC (named Vhc1) is localized to the yeast vacuolar membrane [60], while hNKCC2-GFP was found in the endoplasmic reticulum when expressed in yeast [59]. Determining the subcellular localization of plant CCC proteins in yeast may help to better interpret the results of this functional screen.

## 8. Conclusions

Despite numerous studies across a range of species, the role of CCC proteins in plants is not well defined. Plant and algal CCC proteins form two distinct phylogenetic clades, which are both more closely related to animal KCC than NKCCs. However, the function of proteins from the CCC2 clade, which is present in green algae and moss species, has not been examined yet. There are fewer CCC representatives in the genomes of *Viridiplantae* compared to other large multi-gene membrane protein families (except perhaps moss with seven *CCC* genes), and dicots contain the fewest number of *CCC* genes. Furthermore, knockout of plant genes from the *CCC1* clade in angiosperms has drastic effects on growth and development. The mechanism by which functional disruption of *CCC1* genes confers the multiple aspects of the knockout phenotypes is unclear. Future studies using mutagenized proteins and novel expression systems, to investigate protein domains of interest, are needed to identify structure–function relationships as well as the regulation of plant CCC proteins. Such experiments would allow the reconciliation of CCC membrane localization with protein function, and therefore more clearly establish the purpose of plant CCC.

## Figures and Tables

**Table 1:** Distribution of *CCCs* among selected *Viridiplantae* species with sequenced, annotated genomes.

Common Name	Species	*CCC* per Genome
**Chlorophytes**		
Green algae (freshwater)	*Chlamydomonas reinhardtii*	0
Green algae (marine)	*Ostreococcus lucimarinus*	0
Green algae (marine)	*Ostreococcus tauri*	0
Green algae (marine)	*Micromonas pusilla*	1
Green algae (marine)	*Micromonas* sp. RCC299	1
**Bryophytes**		
Spreading earthmoss	*Physcomitrella patens*	7
Liverwort	*Marchantia polymorpha*	2
**Tracheophytes**		
Selaginella	*Selaginella moellendorffii*	4
Amborella	*Amborella trichopoda*	1
**Monocots**		
Brachypodium	*Brachypodium distachyon*	3
Rice	*Oryza sativa*	2
Sorghum	*Sorghum bicolor*	2
Corn	*Zea mays*	3
**Eudicots**		
Tomato	*Solanum lycopersicum*	2
Potato	*Solanum tuberosum*	2
Grapevine	*Vitis vinifera*	1
Arabidopsis	*Arabidopsis lyrata*	1
Arabidopsis	*Arabidopsis thaliana*	1
Rapeseed	*Brassica rapa*	2
Soybean	*Glycine max*	2
Barrelclover	*Medicago truncatula*	1
Peach	*Prunus persica*	1

## Supplementary Materials

Supplementary Materials can be found at http://www.mdpi.com/1422-0067/19/2/492/s1.
